# Supplementary material for: Human Echinococcosis in the Russian Federation in the 21st Century: A Systematic Review
Source: Microorganisms. 2025 May 14;13(5):1122. doi: 10.3390/microorganisms13051122 (PMC12114422; doi:10.3390/microorganisms13051122)
Supplement: Supplementary file 1 [file microorganisms-13-01122-s001.zip › microorganisms-3592460-supplementary.pdf]

Human echinococcosis in the Russian Federation in the 21st century: a systematic review

Branko Bobić, Tijana Štajner, Vladimir Ćirković, Jelena Srbljanović, Olivera Lijeskić, Neda Bauman, Djordje Zlatković

Supplementary file S1: Figure S1

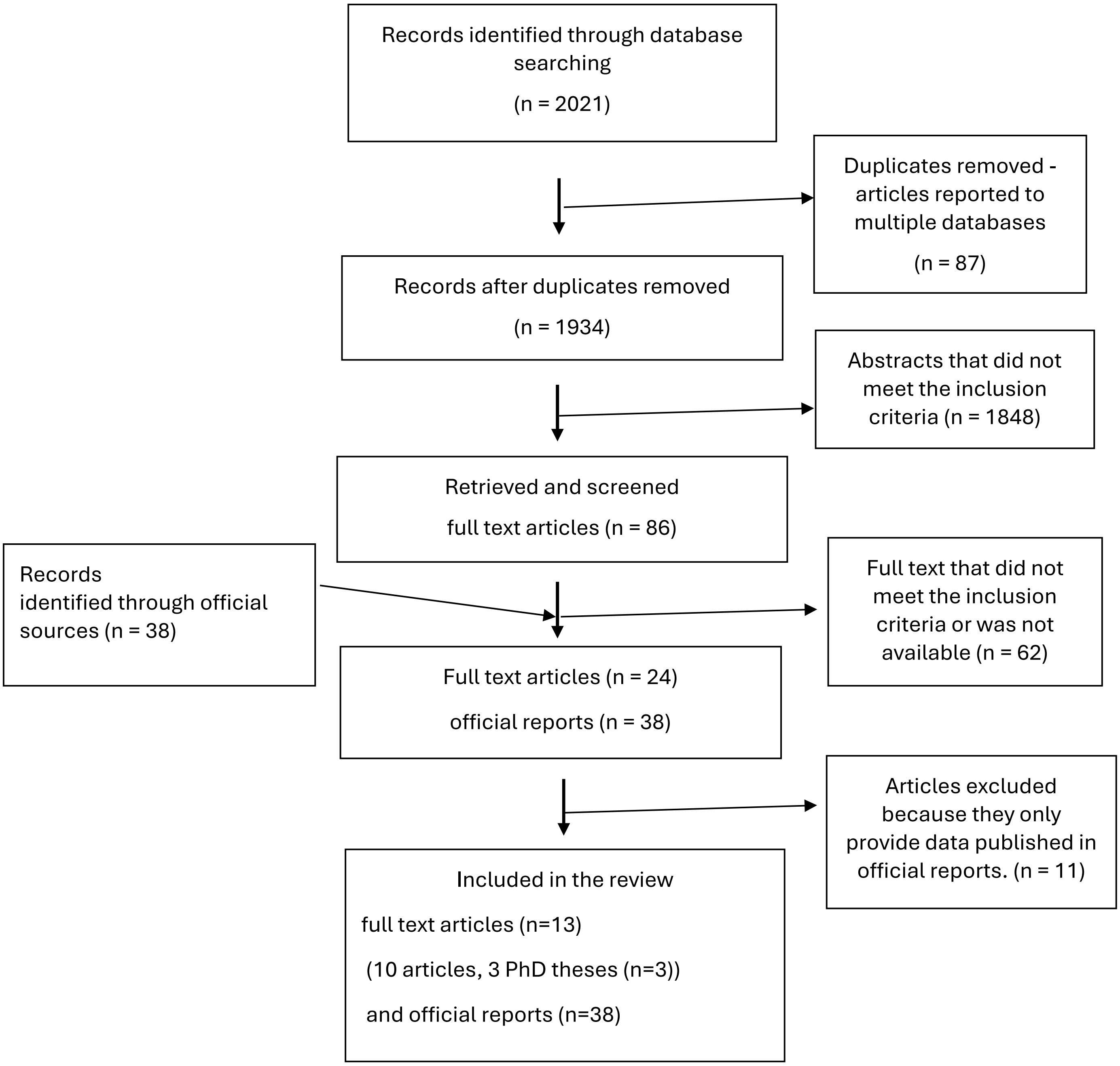

Figure S1. Flow diagram of the database searches

Human echinococcosis in the Russian Federation in the 21st century: a systematic review

Branko Bobić, Tijana Štajner, Vladimir Ćirković, Jelena Srbljanović, Olivera Lijeskić, Neda Bauman, Đorđe Zlatković

Supplementary **file S2: Checklist**

PRISMA 2009 Checklist

	#	Checklist item	Reported on page #
**TITLE**			
Title	1	Identify the report as a systematic review, meta-analysis, or both.	1
**ABSTRACT**			
Structured summary	2	Provide a structured summary including, as applicable: background; objectives; data sources; study eligibility criteria, participants, and interventions; study appraisal and synthesis methods; results; limitations; conclusions and implications of key findings; systematic review registration number.	1
**INTRODUCTION**			
Rationale	3	Describe the rationale for the review in the context of what is already known.	2

Objectives	4	Provide an explicit statement of questions being addressed with reference to participants, interventions, comparisons, outcomes, and study design (PICOS).	2
**METHODS**			
Protocol and registration	5	Indicate if a review protocol exists, if and where it can be accessed (e.g., Web address), and, if available, provide registration information including registration number.	2
Eligibility criteria	6	Specify study characteristics (e.g., PICOS, length of follow-up) and report characteristics (e.g., years considered, language, publication status) used as criteria for eligibility, giving rationale.	3
Information sources	7	Describe all information sources (e.g., databases with dates of coverage, contact with study authors to identify additional studies) in the search and date last searched.	3
Search	8	Present full electronic search strategy for at least one database, including any limits used, such that it could be repeated.	3
Study selection	9	State the process for selecting studies (i.e., screening, eligibility, included in systematic review, and, if applicable, included in the meta-analysis).	3
Data collection process	10	Describe method of data extraction from reports (e.g., piloted forms, independently, in duplicate) and any processes for obtaining and confirming data from investigators.	3
Data items	11	List and define all variables for which data were sought (e.g., PICOS, funding sources) and any assumptions and simplifications made.	3
Risk of bias in individual studies	12	Describe methods used for assessing risk of bias of individual studies (including specification of whether this was done at the study or outcome level), and how this information is to be used in any data synthesis.	NA
Summary measures	13	State the principal summary measures (e.g., risk ratio, difference in means).	NA
Synthesis of results	14	Describe the methods of handling data and combining results of studies, if done, including measures of consistency (e.g., I^2) for each meta-analysis.	NA

Section/topic	#	Checklist item	Reported on page #
Risk of bias across studies	15	Specify any assessment of risk of bias that may affect the cumulative evidence (e.g., publication bias, selective reporting within studies).	NA
Additional analyses	16	Describe methods of additional analyses (e.g., sensitivity or subgroup analyses, meta-regression), if done, indicating which were pre-specified.	
**RESULTS**			
Study selection	17	Give numbers of studies screened, assessed for eligibility, and included in the review, with reasons for exclusions at each stage, ideally with a flow diagram.	3 S1
Study characteristics	18	For each study, present characteristics for which data were extracted (e.g., study size, PICOS, follow-up period) and provide the citations.	NA
Risk of bias within studies	19	Present data on risk of bias of each study and, if available, any outcome level assessment (see item 12).	NA
Results of individual studies	20	For all outcomes considered (benefits or harms), present, for each study: (a) simple summary data for each intervention group (b) effect estimates and confidence intervals, ideally with a forest plot.	NA
Synthesis of results	21	Present results of each meta-analysis done, including confidence intervals and measures of consistency.	NA
Risk of bias across studies	22	Present results of any assessment of risk of bias across studies (see Item 15).	NA
Additional analysis	23	Give results of additional analyses, if done (e.g., sensitivity or subgroup analyses, meta-regression [see Item 16]).	5-11
**DISCUSSION**			
Summary of evidence	24	Summarize the main findings including the strength of evidence for each main outcome; consider their relevance to key groups (e.g., healthcare providers, users, and policy makers).	12-14
Limitations	25	Discuss limitations at study and outcome level (e.g., risk of bias), and at review-level (e.g., incomplete retrieval of identified research, reporting bias).	12-13
Conclusions	26	Provide a general interpretation of the results in the context of other evidence, and implications for future research.	14-15
**FUNDING**			

Funding	27	Describe sources of funding for the systematic review and other support (e.g., supply of data); role of funders for the systematic review.	15

From: Moher D, Liberati A, Tetzlaff J, Altman DG, The PRISMA Group (2009). Preferred Reporting Items for Systematic Reviews and Meta-Analyses: The PRISMA Statement. PLoS Med 6(6): e1000097. doi:10.1371/journal.pmed1000097

For more information, visit: **www.prisma-statement.org**.

Human echinococcosis in the Russian Federation in the 21st century: a systematic review

Branko Bobić, Tijana Štajner, Vladimir Ćirković, Jelena Srbljanović, Olivera Lijeskić, Neda Bauman, Đorđe Zlatković,

Supplementary file S3: Text1

Search protocol

Aim: To collect and analyse epidemiological data on human echinococcosis in the RF in the 2000-2021 period through a systematic review of scientific and grey literature, including official reports

Questions to answer:

The period 2000-2001 was a period of economic recovery in the Russian Federation for which there is insufficient consolidated data on the epidemiological characteristics of human echinococcosis in the new circumstances.

Methods:

The review will be conducted in line with the PRISMA statement 2009 (http://www.bmj.com/content/339/bmj.b2700#alternate) and will include items from the PRIMSA checklist

Articles will be selected for inclusion into the systematic review through identification of all potentially relevant citations implementing the search strategy. Duplicates will be excluded, followed by screening of titles and abstracts with articles excluded if they do not report data of human echinococcosis infection in the RF. Full text articles will be screened for exclusion criteria; those that pass the requirements will be utilised in the review. In addition, the official websites of the Russian government services will be searched, using search phrases, for official reports and reviews and epidemiological bulletins. Full text articles will then be analysed in their entirety and exempted if they only repeated

the epidemiological data already published in the official reports. Relevant data from full-text articles, Phd thesis, and official reports will be extracted and entered into the spreadsheet.

Databases: ,

international databases

PubMed – http://www.ncbi.nlm.nih.gov/pubmed;

Google Scholar – https://scholar.google.com/scholar)

OpenGrey (http://www.opengrey.eu

Russian databases

DVGMU Library, Far Eastern Library of the Medical State University – www.fesmu.ru/elib/Search.aspx?Catalogue=1;

eLIBRARI.RU, Scientific Electronic Library – https://elibrari.ru/;

Ciberleninka – https://cyberleninka.ru/)

Russian Scientific Library, Library of dissertations (http://freereferats.ru/index.php?cat=91&page=14)

Search term:

For international databases: : Echinocoссис OR granulosus OR alveolar OR hydatidosиs AND Russia).

For Russian databases Эхинококк ИЛИ гранулезный ИЛИ альвеолярный ИЛИ хдатидоз AND Россия).

Inclusion:

- Exclusion criteria
- studies concerning other parasites;
- studies conducted outside the R.F.;
- studies that did not include data on human infections;
- studies that did not include data for the study period;

- studies presenting data not related to the epidemiological features of echinococcal infection, but rather, focusing on clinical features, therapy or biology of the parasite;
- studies containing a general overview of the topic without original data;
- studies presenting duplicate data.
- Languages: All
- Year of data collection: 1st January 2000 – 1st December 2021
- Geographical range: Russian Federation

Human echinococcosis in the Russian Federation in the 21st century: a systematic review

Branko Bobić, Tijana Štajner, Vladimir Ćirković, Jelena Srbljanović, Olivera Lijeskić, Neda Bauman, Đorđe Zlatković

Supplementary file S4: Table S2

Table S2. Analyzed reports of human echinococcosis in the Russian Federation

Source	Ref	Document tipe	Addition Information
Infection and immunity 2012, T. 2, No. 1–2 350-351	15	article	data on human echinococcosis
Theory and practice of combating parasitic diseases. 2011. No. 12	18	article	data on human echinococcosis
Lechaschi Vrach. 2023; 3 (26): 58-63.	19	article	data on humane chinococcosis
PH&LE, 2017, 12: 51-56.	20	article	data on hum anechinococcosis
Theory and practice of combating parasitic diseases International scientific conference May 19–21, 2021, Moscow Collection of scientific articles Issue 22 162-167	14	article	data on human echinococcosis
Doctor.Ru. 2018. No. 7 (151):35-38	25	article	data on human echinococcosis
Theory and practice of combating parasitic diseases. 2023. 2, 177-183		article	data on human echinococcosis
Epidemiology and Vaccinal Prevention. 2017;16(1):43-46.		article	data on human echinococcosis
Dissertation Orenburg State Medical University, Orenburg 2015.	17	Phd thesis	data on human echinococcosis
Doctoral thesis Sechenov University, Moscow–2020	127	Phd thesis	data on hum anechinococcosis
Dissertation, Samara State Medical Uниversity, Samara, 2020	26	Phd thesis	data on human echinococcosis
Federal Service for Surveillance on Consumer Rights Protection and Human Wellbeing (Rospotrebnadzor) letter: N 01/7782-16-27	13	article	data on human echinococcosis
Federal Service for Surveillance on Consumer Rights Protection and Human Wellbeing (Rospotrebnadzor) letter: № 01/14780-13-32	23	article	data on humane chinococcosis

Rosselkhoznadzor, - Federal Service for Veterinary and Phytosanitary Surveillance. Information-Analytical Center. Epizootic situation in the Russian Federation annual reports 2008-2021	21	official annual reports	data on humane chinococcosis
Federal Service for Surveillance on Consumer Rights Protection and Human Wellbeing (Rospotrebnadzor) Annual state reports 2008-2022	14	official annual reports	data on humane chinococcosis
Service for Surveillance on Consumer Rights Protection and Human Wellbeing (Rospotrebnadzor) department in Moscow annual reports 2011-2021	16	official annual reports	data on humane chinococcosis
Rosstat -Federal State Statistics Service of the Russian Federation. Russian Statistical Yearbook 2003-2023. https://rosstat.gov.ru/folder/210/document/12994Rosstat	21	official reports	demographic data
Rosstat (Federal State Statistics Service). Regions of Russia. Socio-economic indicators – 2014.		official report	Economic indicators
